# Generating mouse models for biomedical research: technological advances

**DOI:** 10.1242/dmm.029462

**Published:** 2019-01-08

**Authors:** Channabasavaiah B. Gurumurthy, Kevin C. Kent Lloyd

**Affiliations:** 1 Developmental Neuroscience, Munroe Meyer Institute for Genetics and Rehabilitation, University of Nebraska Medical Center, Omaha, NE 68106-5915, USA; 2 Mouse Genome Engineering Core Facility, Vice Chancellor for Research Office, University of Nebraska Medical Center, Omaha, NE 68106-5915, USA; 3 Department of Surgery, School of Medicine, University of California, Davis, CA 95618, USA; 4 Mouse Biology Program, University of California, Davis, CA 95618, USA

**Keywords:** CRISPR, Genome editing, Mouse, Mutagenesis

## Abstract

Over the past decade, new methods and procedures have been developed to generate genetically engineered mouse models of human disease. This At a Glance article highlights several recent technical advances in mouse genome manipulation that have transformed our ability to manipulate and study gene expression in the mouse. We discuss how conventional gene targeting by homologous recombination in embryonic stem cells has given way to more refined methods that enable allele-specific manipulation in zygotes. We also highlight advances in the use of programmable endonucleases that have greatly increased the feasibility and ease of editing the mouse genome. Together, these and other technologies provide researchers with the molecular tools to functionally annotate the mouse genome with greater fidelity and specificity, as well as to generate new mouse models using faster, simpler and less costly techniques.

## Abstract (precis)

**Summary:** Newer molecular technologies to precisely and efficiently manipulate the mammalian genome are enabling the production of more scientifically valuable animal models.

## Introduction

Researchers are entering a new era of human disease modeling in animals. For many years now, the laboratory mouse (*Mus musculus*) has remained the quintessential research animal of choice for studying human biology, pathology and disease processes (Rosenthal and Brown, 2007; Lloyd et al., 2016). The mouse possesses numerous biological characteristics that make it the most commonly used animal in biomedical research for modeling human disease mechanisms; these characteristics include its short life cycle, gestation period and lifespan, as well as its high fecundity and breeding efficiency (Silver, 2001). Another key advantage is its high degree of conservation with humans, as reflected in its anatomy, physiology and genetics (Justice and Dhillon, 2016).

**Figure:**
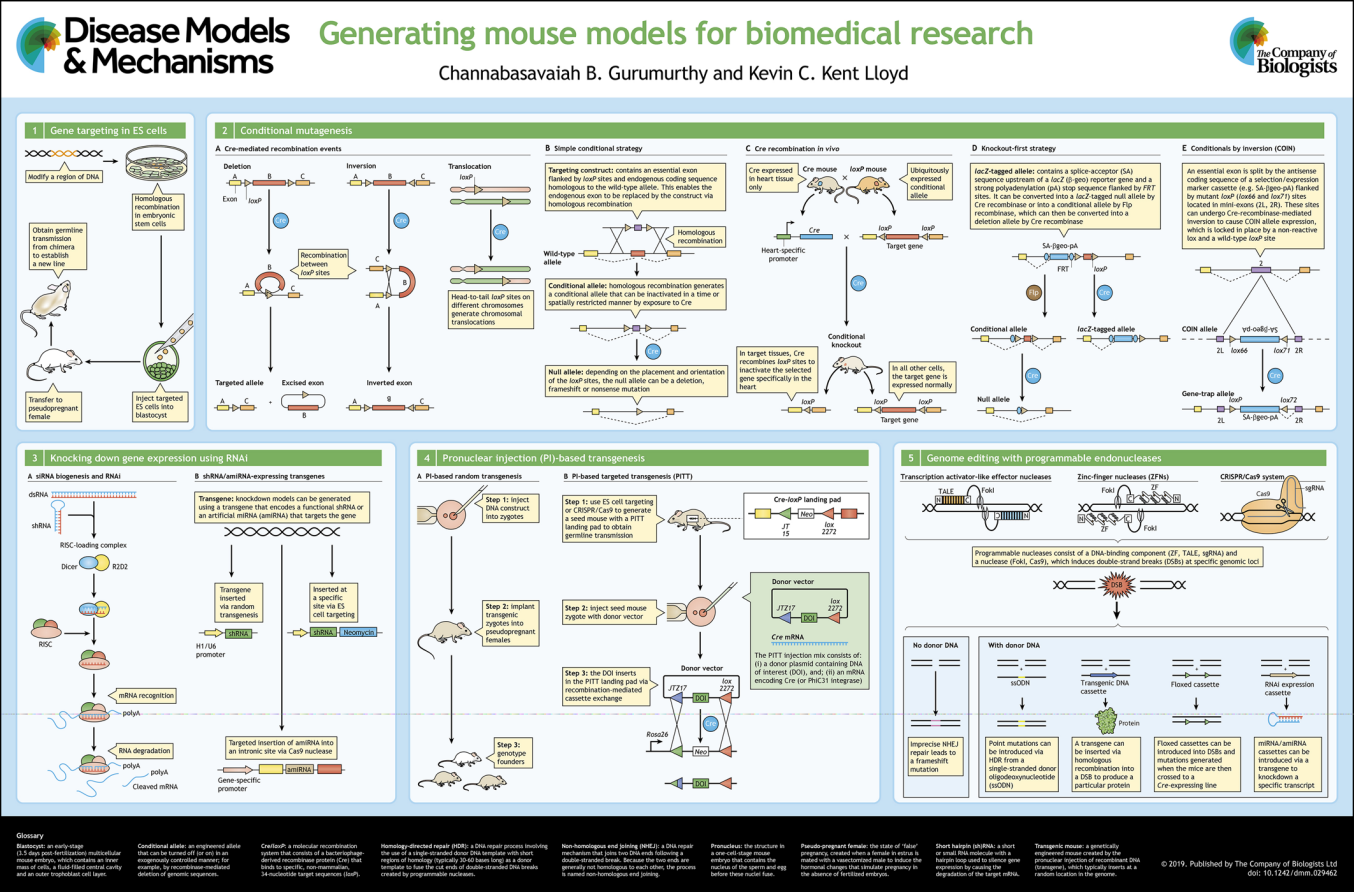

The highly conserved genetic homology that exists between mice and humans has justified the development of technologies to manipulate the mouse genome to create mouse models to reveal the genetic components of disease. It is important to note that, as technologies for genetic engineering and phenotypic analysis have advanced, some studies using mouse models have struggled to accurately predict human disease pathogenesis and clinical response to drug therapy (Perrin, 2014). For these reasons, it is essential to apply scientific principles of rigor and reproducibility (Kilkenny et al., 2010; Karp et al., 2015) when designing and conducting experiments to associate mouse genes with human phenotypes at a systems level (Perlman, 2016).

Early mouse genetics research relied on mice having visible physical defects and readily measurable phenotypes, such as those caused by random spontaneous or induced mutations (Russell et al., 1979; Justice, 1999). This ‘forward genetics’ approach depends on the presence of a phenotype to guide the search for the underlying genetic mutation. With the advent of techniques that enabled molecular cloning and the use of recombinant DNA to efficiently manipulate mouse genomes, researchers no longer needed to search for a relevant phenotype. Instead, they could engineer a pre-determined specific mutation into the mouse genome in real time in pluripotent mouse embryonic stem (ES) cells (Gordon and Ruddle, 1981; Gordon et al., 1980; Palmiter et al., 1982; Thomas and Capecchi, 1986, 1987). This ‘reverse genetics’ approach enabled scientists to study the phenotypic consequences of a known specific genetic mutation. This approach can generate ‘knockout’ mice (see Box 1 for a glossary of terms) by genetically manipulating the genome of ES cells, and then injecting the targeted cells into morulae or blastocysts (Box 1), which are then implanted into pseudopregnant female mice (Box 1). The resulting chimeric embryos develop into offspring that bear the desired gene deletion. After backcrossing to test for germline transmission of the knockout allele and subsequent intercrossing to achieve homozygosity, the phenotypic consequences of the mutation can be assessed. Phenotypes can also be assessed in transgenic mice (Box 1), which are generated by introducing an exogenous gene via microinjection into the one-cell-stage zygote. When successful, these genetic manipulations can also undergo germline transmission to the next generation (Palmiter et al., 1982; Brinster et al., 1989).

With the sequencing of the mouse and human genomes (Venter et al., 2001; Mouse Genome Sequencing Consortium, 2002), attention soon turned to determining the function of protein-coding genes (Nadeau et al., 2001). A growing number (∼6000) of inherited disease syndromes (https://www.omim.org/statistics/geneMap) further motivated efforts to functionally annotate every human gene and to determine the genetic basis of rare, simple and common complex human diseases using mouse models. Mouse models are thus vitally important for elucidating gene function. Those that express the pathophysiology of human disease are an essential resource for understanding disease mechanisms, improving diagnostic strategies and for testing therapeutic interventions (Rosenthal and Brown, 2007; Bradley et al., 2012; Justice and Dhillon, 2016; Meehan et al., 2017). Even mouse models that only partially recapitulate the human phenotype, such as mutations in individual paralogs, can still provide important insights into disease mechanisms.

In this At a Glance article, we review recent technological advances for generating new and improved mouse models for biomedical research. This article aims to update a previous poster published in this journal several years ago (Justice et al., 2011). This earlier article discussed the role of natural variation, random transgenesis, reverse genetics via ES-cell-derived knockouts, forward genetics via ethylnitrosurea (ENU)-induced chemical mutagenesis, and genetic manipulation using transposons in the generation of mouse models. Many technological advances have since emerged, leading to refinements and improvements in the generation of more precise mouse models. These new technologies overcome some of the limitations of earlier mouse models by adding specificity, reproducibility and efficiency to the generation of alleles that can expand our knowledge of disease pathogenesis. For example, the ability to generate mouse models that recapitulate the single-nucleotide variants (SNVs) found in humans will enable us to differentiate between disease-causing and disease-associated mechanisms (Hara and Takada, 2018).

In the poster accompanying this article, we feature four areas of advancement:

(1) conditional mutagenesis strategies in mouse ES cells;

(2) gene function knockdown using RNA interference (RNAi);

(3) targeted transgenesis in zygotes (Piedrahita et al., 1999; Shen et al., 2007) via homologous recombination (Box 1) in ES cells; and

(4) the use of programmable endonucleases (Box 1) in zygotes, to edit and manipulate the mouse genome in ways not previously possible.

These technologies represent a new paradigm in our ability to manipulate the mouse genome. However, as we discuss, these approaches are not without limitations. For example, the success of conditional mutagenesis can be hampered by poor gene-targeting efficiency in ES cells and by the limited production of germline-competent chimeras (Box 1) that can transmit the mutant allele to subsequent generations in their germline. Furthermore, protein expression can be highly variable following mRNA knockdown by RNAi, which can make experimental reproducibility a challenge. The major limitations of programmable endonucleases, the latest genome-editing tools, is mosaicism and their potential, albeit addressable, problem of inducing off-target mutations. Nonetheless, such pitfalls do not detract from the versatility that these newer technologies afford for manipulating the mouse genome.

**Box 1. Glossary**

**Blastocyst:** an early-stage (3.5 days post-fertilization) multicellular mouse embryo, which contains an inner mass of cells, a fluid-filled central cavity and an outer trophoblast cell layer.

**Chimera:** a founder mouse that contains a mix of gene-targeted, embryonic stem (ES)-cell-derived cells and host blastocyst-derived cells, typically identified by the contribution of the two different genetic backgrounds of somatic cells to its coat color.

**Conditional alleles:** an engineered allele that can be turned off (or on) in an exogenously controlled manner; for example, by recombinase-mediated deletion of genomic sequences.

**Cre/*loxP*:** a molecular recombination system that consists of a bacteriophage-derived recombinase protein (Cre) that binds to specific, non-mammalian, 34-nucleotide target sequences (*loxP*).

**Footprint-free point mutations:** an induced mutation that is created without changes being made to untargeted sequences and without leaving exogenous DNA in place.

**Gene targeting:** the methods used to make sequence changes to a specific gene rather than making random sequence changes; for example, gene targeting can be used to inactivate a gene.

**Homologous recombination:** a natural DNA recombination process that occurs, for example, during meiosis and DNA repair, in which similar or identical DNA sequences are exchanged between two adjacent strands of DNA.

**Homology-directed repair (HDR):** a DNA repair process involving the use of a single-stranded donor DNA template with short regions of homology (typically 30-60 bases long) as a donor template to fuse the cut ends of double-stranded DNA breaks created by programmable nucleases.

**Knock-down mouse:** a genetically altered mouse in which gene expression is lowered or silenced by using RNAi to degrade the mRNA of that gene.

**Knock-in mouse:** a genetically altered mouse in which a new mutation is introduced into an endogenous gene or an exogenous gene is introduced using genetic-engineering technologies.

**Knockout mouse:** a genetically altered mouse in which an endogenous gene is deleted and/or inactivated using genetic-engineering technologies.

***loxP*-stop-*loxP*:** a commonly used DNA cassette, containing a stop codon flanked by *loxP* sites, included between the promoter and the coding sequences, to prevent expression of the coding sequence until the stop codon is excised by Cre-mediated recombination.

**Morula:** an early-stage (2.5 days post-fertilization) pre-implantation mouse embryo, typically consisting of 4-8 blastomeres.

**Non-homologous end joining (NHEJ):** a DNA repair mechanism that joins two DNA ends following a double-stranded break. Because the two ends are generally not homologous to each other, the process is named non-homologous end joining.

**Programmable endonuclease:** an enzyme that, when coupled with molecular targeting elements (e.g. a guide RNA), creates site-specific double-stranded DNA breaks.

**Pronuclei:** the structure in a one-cell-stage mouse embryo that contains the nucleus of the sperm and egg before these nuclei fuse.

**Pseudopregnant female:** the state of ‘false’ pregnancy, created when a female in estrus is mated with a vasectomized male to induce the hormonal changes that simulate pregnancy in the absence of fertilized embryos.

**Recombinase-mediated cassette exchange (RMCE):** a DNA integration strategy that uses site-specific recombinases, such as Cre or Flp, to exchange a DNA segment from one DNA molecule to another. Both the donor and target sequence are flanked by site-specific recombination sites, such as *loxP* or *FRT*. Double reciprocal recombination between these sites brings about DNA exchange.

**Safe-harbor sites:** a genomic locus that, when genetically manipulated, neither interferes with the expression of an integrated transgene nor disrupts endogenous gene activity.

**Short hairpin (sh)RNA:** a short or small RNA molecule with a hairpin loop used to silence gene expression by causing the degradation of the target mRNA.

**Small interfering (si)RNA:** a short or small linear RNA molecule used to interfere with, or to silence, gene expression by causing the degradation of the target mRNA.

**Transgenic mouse:** a genetically engineered mouse created by the pronuclear injection of recombinant DNA (transgene), which typically inserts at a random location in the genome.

## Conditional mutagenesis strategies in mouse ES cells

The most common form of mouse genetic manipulation is the creation of gene knockout models. Gene-targeting in mouse ES cells was pioneered in the late 1980s and was first used to generate ubiquitous knockout models, in which the gene is deleted in every cell (Thomas and Capecchi, 1987; Thompson et al., 1989). We refer readers to the previous At a Glance article on modeling human disease in mice (Justice et al., 2011) for details on how to use gene targeting (Box 1) to generate simple deletion and/or conditional alleles (Box 1) in ES cells to generate whole-body and tissue-specific knockout mice, respectively. In this article, we focus on the generation of more-complex alleles in ES cells (Poster panel 1) that retain wild-type expression and are amenable to conditional, tissue-specific and/or time-dependent deletion. This approach is particularly necessary for manipulating the approximately 30% of genes that affect the viability of homozygous mutants when deleted (Dickinson et al., 2016). For example, embryonic lethality caused by the deletion of the coding regions of *Mixl1* (Pulina et al., 2014), *Erbb4* (Gassmann et al., 1995) or *Brca1* (Xu et al., 1999) can be rescued by conditional mutagenesis. This generates models that can be used to investigate specific gene-dependent processes during mammalian embryogenesis (Pulina et al., 2014), neurodevelopment (Golub et al., 2004) and breast cancer (Shakya et al., 2008) when combined with an appropriate *Cre*-expressing line that enables tissue- or developmental-stage-specific gene deletion (Dubois et al., 2006).

The versatility of naturally occurring recombinase-enzyme–target-sequence systems, such as Cre*/loxP* (Box 1) and Flp*/FRT*, which derive from bacteria and yeast, respectively, have been adapted to create tools for manipulating mammalian genomes (Gu et al., 1994; Rajewsky et al., 1996; Dymecki, 1996). These tools have dramatically expanded the types and varieties of alleles that can be designed to study gene function *in vivo* (Dymecki, 1996; Nagy, 2000; Nern et al., 2011). A fundamental principle of conditional mutagenesis is the ability to efficiently and reliably convert a functional allele into a mutant one in a specific cell type (called tissue-specific conditional mutagenesis) and/or at a specific time point during development (called time-specific or ‘inducible’ conditional mutagenesis).

Numerous strategies using recombinase-enzyme–target-sequence systems have been developed for conditional mutagenesis (Marth, 1996). Common to all these strategies is the use of short palindromic recombinase target sequences to flank a specific region of a gene (e.g. a critical coding exon common to all transcripts). Such sequences include the Cre*-*associated *loxP* sequence (to generate a ‘floxed’ allele) or the Flp-associated *FRT* sequence (to generate an ‘*FRT*’-flanked allele) (Bouabe and Okkenhaug, 2013). In the absence of the associated recombinase enzyme, these flanking sequences have no effect on normal transcription nor on the expression of the endogenous gene. However, when exposed to the recombinase, the flanking recombinase target sequences recombine with each other to excise or invert the critical coding exon, depending on their orientation and positioning (McLellan et al., 2017) (Poster panel 2A). In its simplest use, if two flanking recombinase target sequences are placed in an asymmetrical head-to-tail orientation, they will recombine to delete the intervening genetic sequence upon exposure to recombinase. Alternatively, if pairs of target sequences are positioned symmetrically in a head-to-head orientation, their recombination will invert the intervening sequence. If target sequences are located on different chromosomes, recombination results in a chromosomal translocation.

There are different ways to elicit recombination. For example, as shown in Poster panel 2B, when a mouse that expresses a floxed allele is mated with a transgenic mouse that expresses the recombinase gene, its progeny will express the recombined allele (Gu et al., 1994). The tissue(s) in which the allele is recombined will depend on the expression pattern of the recombinase, i.e. where the promoter is activated to drive tissue-specific expression of the recombinase. Recombination can also be induced by the *in vitro* treatment of embryos or tissues with cell-permeable recombinase protein, or via the delivery of viral vectors that express the recombinase (Chambers et al., 2007; Lewandoski et al., 1997; Su et al., 2002). Recombinase activity can also be targeted to particular tissues by driving the expression of a recombinase from a cell-specific promoter. Recombinase expression can also be induced by expressing the recombinase from an inducible (e.g. drug-responsive) promoter (Sauer, 1998).

The simplest example of the recombinase-enzyme–target-sequence system is shown in Poster panel 2C. This panel shows a molecular targeting construct in which the critical coding exon is flanked by *loxP* sites. The construct also contains a contiguous endogenous coding sequence of between 3 and 8 kb that is homologous to the wild-type allele. This construct is then introduced into ES cells, for example by electroporation, where it then replaces, via homologous recombination, the endogenous wild-type allele (Hadjantonakis et al., 2008). The conditional allele can then undergo recombination upon exposure to the recombinase to delete the intervening critical coding exon, thereby inhibiting gene expression (null allele).

Another strategy, termed ‘knockout-first’, uses a variation of gene targeting to create a highly versatile allele that combines both gene trap (Friedel and Soriano, 2010) and conditional gene targeting (Jovicić et al., 1990) to generate a *lacZ*-tagged knockout allele (Testa et al., 2004) (Poster panel 2D). The ‘knockout-first’ allele is generated by inserting an *FRT*-flanked gene-trap vector, which contains a splice-acceptor sequence upstream of a *lacZ* reporter gene and a strong polyadenylation stop sequence, into an upstream intron. This creates an in-frame fusion transcript that will disrupt the expression of the targeted allele. Additionally, an adjacent exon coding sequence is flanked with *loxP* sites (Rosen et al., 2015). This allele can then be converted into a null allele by Cre to abrogate gene expression or into a conditional allele by Flp, which can subsequently be converted by Cre into a null allele (Testa et al., 2004; Skarnes et al., 2011). The knockout-first strategy is versatile because it uses a single targeting vector to monitor gene expression using *lacZ* and tissue-specific gene function using Cre, thereby avoiding embryonic lethality. This strategy has been used effectively to enable the rapid and high-throughput production of thousands of gene knockouts in mouse ES cells in large-scale, genome-wide targeted mutagenesis programs, such as the International Knockout Mouse Consortium (IKMC) (Bradley et al., 2012). Hundreds of mutant mouse models of human genetic diseases have been generated using the knockout-first strategy, including models of skin abnormalities (Liakath-Ali et al., 2014), bone and cartilage disease (Freudenthal et al., 2016), and age-related hearing loss (Kane et al., 2012).

Lastly, an elegant technique termed ‘conditionals by inversion’ (COIN) employs an inverted COIN module that contains a reporter gene (e.g. *lacZ*) flanked by mutant recombinase target sites (*lox66* and *lox71*) positioned in a head-to-head orientation to enable inversion by Cre recombinase (Albert et al., 1995) inserted into the anti-sense strand of a target gene (Economides et al., 2013) (Poster panel 2E). Cre ‘flips’ the COIN module into the sense strand, interfering with and inhibiting target-gene transcription while activating the reporter. The COIN approach is particularly applicable to single-exon genes and to genes in which the exon–intron structure is not clearly defined. This approach has been used to model an angiogenesis defect in delta-like 4 (*Dll4*) knockout mice (Billiard et al., 2012) and to generate immunological phenotypes in interleukin 2 receptor, gamma chain (*Il2rg*) knockout mice (Economides et al., 2013).

## Gene expression knockdown using RNAi

About two decades ago, researchers observed that the introduction of double-stranded RNA (dsRNA) that was homologous to a specific gene resulted in its posttranscriptional silencing (Fire et al., 1998). This dsRNA-induced gene silencing was termed RNA interference (RNAi), and it occurs via two main steps (Poster panel 3A). First, Dicer, an enzyme of the RNase III family of nucleases, processes the dsRNA into small double-stranded fragments termed siRNAs (small interfering RNAs; Box 1). Then, the siRNAs are incorporated into a nuclease complex called RISC (for RNA-induced silencing complex), which unwinds the siRNA and finds homologous target mRNAs using the siRNA sequence as a guide; this complex then cleaves the target mRNAs. In the early 2000s, some groups explored whether RNAi could be used to reduce (or ‘knock down’) gene expression in mice by creating transgenic mice that express siRNA (Poster panel 3B). The first proof-of-principle for gene knockdown was demonstrated by delivering lentivirus particles expressing siRNA into green fluorescent protein (GFP) transgenic mice to knock down GFP (Tiscornia et al., 2003). Subsequently, knockdown mice were generated using standard pronuclear injection of constructs that express short-hairpin RNAs (shRNA; Box 1) (Chang et al., 2004; Peng et al., 2006; Seibler et al., 2007; Dickins et al., 2007). Some examples of transgenic knockdown disease models include: an *Abca1*-deficient mouse line that mimics Tangier disease (Chang et al., 2004); insulin receptor (*Insr*)-knockdown mice that develop severe hyperglycemia within 7 days (Seibler et al., 2007); and the reversible knockdown of *Trp53* as a model useful for tumor regression studies (Dickins et al., 2007).

The advantage of the RNAi knockdown strategy over traditional methods for generating knockout mice is that it provides a rapid and inexpensive approach by which to selectively and, in some cases, reversibly block the translation of a transcript. Although knockdown models can be generated more quickly and cheaply than gene-targeted knockout models (Liu, 2013), a key disadvantage of a knockdown is that transcript inhibition can be variable and transient, and therefore less reliable and reproducible than a knockout. The effects of random insertion, together with varying levels of RNAi in different cells within a tissue, were among the most common pitfalls associated with using RNAi technology to modify mouse gene expression (Peng et al., 2006; Yamamoto-Hino and Goto, 2013).

Because of such challenges, and due to the lack of success in generating reliable transgenic RNAi models, this approach did not gain the expected popularity. Alternative strategies were developed to overcome the effect of randomly inserted RNAi constructs by targeting the knockdown cassettes to safe-harbor sites (Box 1), such as the *Gt(ROSA)26Sor* locus (Kleinhammer et al., 2010) or the *Cola1* locus (Premsrirut et al., 2011). These strategies also include making the system modular by incorporating features such as: (i) the Flp-*FRT* recombinase-mediated cassette exchange (RMCE; Box 1), which facilitates the insertion of a single-copy expression cassette; (ii) a fluorescence reporter that enables gene expression analysis; (iii) microRNA (miRNA) architectures, such as miR30 with reduced general toxicity (McBride et al., 2008); and (iv) tetracycline-inducible elements to enable the expression of the RNAi cassettes upon doxycycline administration (Chang et al., 2004; Seibler et al., 2007). A few models that are useful for cancer research have been generated using these approaches, such as *Pax5* and *eIF4F* knockdown models for leukemia (Lin et al., 2012; Liu et al., 2014). However, interest in generating knockdown models, as well as in using ES-cell-based gene targeting, began to wane with the development of programmable nuclease technologies (as discussed later).

More recently, an elegant approach that combines the use of the RNA-guided Cas9 nuclease system with RNAi technology has been developed to generate knockdown mouse models by inserting the knockdown cassettes into the intronic sites of endogenous genes (Miura et al., 2015). With this method, a single-copy artificial miRNA against the *Otx2* gene was inserted into intron 6 of the *Eef2* gene to knock down *Otx2* in mid-gestation mouse embryos. This strategy was also used to conditionally activate knockdown cassettes using unidirectional recombinase-mediated inversion of the shRNA cassette. The Miura et al. method offers a feasible and simple strategy to generate gene knockdown models because: (i) it uses an endogenous promoter, unlike other knockdown approaches that require an exogenous promoter to drive the RNAi cassette; (ii) the knockdown cassette is inserted as a single copy at a known site in the genome, unlike approaches that randomly insert the cassette with no control over the number of copies inserted or the number of genomic insertion sites; and (iii) the transgene is not susceptible to silencing, in contrast to other transgenes that are often silenced following random genomic integration.

## Pronuclear injection-based transgenesis

Traditional transgenic methods developed over three decades ago involve the injection of linearized DNA expression cassettes into fertilized zygotes (Gordon et al., 1980; Palmiter et al., 1982) (Poster panel 4A). Some of the most commonly used transgenic DNA expression cassettes include: (i) cDNA encoding the wild-type or mutant allele; (ii) inducible reporter cassettes, such as the *loxP*-stop-*loxP* reporter (Box 1), that incorporate markers such as *lacZ* or the fluorescent reporters GFP, red fluorescent protein (RFP) or tdTomato; (iii) recombinases, such as Cre (Gu et al., 1994), tamoxifen-inducible Cre (CreERT2) (Feil et al., 1996) and Flp (Dymecki, 1996); and (iv) transcriptional inducers, such as tetracycline transactivators (*tTA*) or reverse tetracycline transactivators (*rtTA*) (Gossen and Bujard, 1992).

To produce transgenic mice, a DNA construct is microinjected into the pronuclei (Box 1) of one-cell-stage zygotes (Bockamp et al., 2008). All or part of the injected DNA then inserts randomly at one or more genomic loci as either a single or as multiple (e.g. tandem-repeat) copies. The suitability of this approach for generating animal models is limited by the uncertainty of obtaining a desired level of gene expression due to the random nature of transgene insertion and copy number (Chiang et al., 2012). As a result, ES-cell-based methods were developed to target expression cassettes (such as those encoding Cre) into a specific locus in the genome; for example, the *Gt(ROSA)26Sor* locus, which enables the ubiquitous expression of an inserted transgene (Soriano, 1999). Depending on the construct and insertion site, transgene expression could be driven by a target gene's endogenous promoter and/or by other regulatory elements (Rickert et al., 1997). In this way, an intact, single-copy transgene becomes integrated into a predetermined genomic location in ES cells via homologous recombination, thereby optimizing transgene expression (Rickert et al., 1997; Soriano, 1999). The targeted ES cells are then introduced into morulae or blastocysts, as previously explained, before being implanted into pseudopregnant females. Although this approach overcomes some of the constraints inherent to random transgenesis (such as high variability of gene expression, and difficulty in obtaining the desired transgene expression patterns and levels), homologous recombination has technical hurdles of its own that make it expensive, labor intensive and time consuming. In addition, germline transmission of the exogenous allele can fail, creating a frustrating struggle for researchers who need to reliably and regularly manipulate the mouse genome (Ohtsuka et al., 2012a). Another disadvantage of the ES cell targeting approach is that ES cell genomes do not always remain stable in culture, and can undergo changes before and after gene targeting (Liang et al., 2008).

The recently developed targeted transgenic technologies enable the integration of single-copy transgenes at specific loci in the genome, directly via pronuclear injection. In pioneering work, Masato Ohtsuka and co-workers developed a method called pronuclear injection-based targeted transgenesis (PITT) (Ohtsuka et al., 2010), which allows a single copy of a complete transgene to be precisely inserted at a desired genomic locus in the zygote (Poster panel 4B). The PITT method involves two steps. First, a landing pad (for example, a cassette containing a combination of mutant *loxP* sites) is inserted at a defined locus in ES cells to generate a ‘seed’ mouse strain. Second, the PITT components – a donor plasmid containing the DNA of interest (DOI) and a Cre source (either plasmid or mRNA) – are injected into fertilized eggs collected from the seed strain mice. The DOI inserts at the landing pad via recombination-mediated cassette exchange (RMCE). The landing pad and the donor DNA contain compatible sequence elements that enable the donor DNA to insert precisely into the target locus. In the first report (Ohtsuka et al., 2010), the authors employed a well-established Cre*-loxP* system (as the components of the landing pad and the donor plasmid elements) to achieve RMCE. Soon after the first description of the PITT technology, another group reported a similar approach using the PhiC31 integrase and *attP/B* system, which correspond to the landing pad components and donor plasmid elements (Tasic et al., 2011). This modified method to achieve targeted transgenesis was named Targatt™ (Chen-Tsai et al., 2014). The main advantages of the various targeted transgenesis methods that use either Cre*-loxP* recombination or PhiC31*-attP/B* integration, are that: (i) they overcome the problems associated with random transgene insertion, such as fragmented insertion of the transgenes, multicopy insertions, transgene silencing or interference in the expression of the endogenously disrupted gene; and (ii) they resolve the time and cost limitations associated with ES-cell-based approaches by targeting DNA cassettes to specific sites in the genome.

In initial reports of the PITT method, the Cre recombinase was encoded by a plasmid, and the plasmid DNA was injected into the pronuclei of zygotes together with the donor DNA. This method has since been improved by: (i) the use of *Cre* mRNA instead of plasmid DNA, which was done because plasmid DNA needs to be transcribed, which delays the expression of Cre, by which time the donor DNA might have degraded (Ohtsuka et al., 2012b); (ii) the development of new PITT-compatible donor vectors (Ohtsuka et al., 2012b); and (iii) the development of a seed mouse strain that contains both Cre*-loxP* and PhiC31-*attP/B* cassette insertion systems, providing researchers with the flexibility to use either (Ohtsuka et al., 2015). In this format, multiple different PITT donor plasmids can be included in the microinjection mix: any one of these donors can be inserted at the landing pad in separate founder mice, resulting in independent transgenic mouse lines generated in a single session of microinjection. These latest technical tools, dubbed ‘improved PITT’ (*i-*PITT), allow up to three transgenic mouse lines to be generated simultaneously, such that each line has a different DOI after a single microinjection session (Ohtsuka et al., 2015). The PITT technology is reviewed in detail in Ohtsuka et al., 2012a and a comprehensive list of available PITT tools was recently described (Schilit et al., 2016). The PITT/*i*-PITT approaches have been used to generate many reliable single-copy transgenic reporter mouse lines that are useful for disease research, including in neuroscience (Madisen et al., 2015) and nephrology (Tsuchida et al., 2016). For example, Tsuchida et al. (2016) reported generating a nephrin-promoter-driven EGFP transgenic mouse model; they further showed that cultured glomeruli from this model serve as tools to screen for compounds that enhance nephrin-promoter activity. Although PITT strategies have overcome the limitations of random transgenesis, a major pitfall of this approach is that custom PITT seed mouse strains need to be generated for a given locus and maintained as breeder colonies as zygote donors for targeted transgenesis.

Despite the technical advances in genetic engineering over the past four decades, one recent and remarkable technical breakthrough is rapidly superseding nearly all of these advances: programmable endonucleases.

## Programmable endonucleases for genome editing

Programmable endonucleases bypass the classical ES-cell-based gene-targeting steps to engineer a precise and heritable mutation at a specific site in the genome. Injection directly into one- or two-cell-stage embryos enables the germline modification of a specific genetic locus without the need for the three complex steps above.

Programmable endonucleases can introduce genetic mutations in one of two ways (Joung and Sander, 2012; Gaj et al., 2013; Sander and Joung, 2014; Cox et al., 2015). They can cause: (i) imprecise, error-prone DNA repair as a result of non-homologous end joining (NHEJ; Box 1) of the cleaved DNA ends; or (ii) the precise repair of cleaved DNA ends by homology-directed repair (HDR; Box 1) via the co-injection of a DNA repair template. Nonetheless, the imprecise insertion of the donor DNA can still occur in HDR-mediated repair. The development of programmable endonucleases for genome editing has opened up a whole new set of technical possibilities to create animal models for biomedical research using virtually any suitable species.

There are four major platforms that employ programmable endonucleases, which were initially discovered in microbiology research applications (Chevalier and Stoddard, 2001; Li et al., 1992; Mojica and Garrett, 2013; Mojica et al., 1993; Römer et al., 2007) and have since been repurposed for editing the genomes of higher animals, including mice. They are, in the order they were developed: homing endonucleases (HEs); zinc-finger nucleases (ZFNs); transcription activator-like effector nucleases (TALENs); and the clustered regularly interspaced short palindromic repeats/CRISPR-associated 9 (CRISPR/Cas9) system (Poster panel 5). Common to all four programmable endonuclease platforms is their sequence-specific nuclease activity, which allows researchers to cleave DNA at a specific target site for genome editing (Joung and Sander, 2012; Gaj et al., 2013; Sander and Joung, 2014; Cox et al., 2015).

The HEs were among the first of the endonucleases (Rouet et al., 1994) to be used for genome manipulation. Although HEs were shown to increase gene-targeting efficiency in ES cells (Smih et al., 1995), there is little evidence to suggest that they have been used successfully to genetically engineer mutant mice. This is probably because of the numerous steps required to design and construct HEs to target specific genomic sites, and because only a small number of genomic sites could be targeted. The ZFNs, unlike HEs, offered greater flexibility as they are easier to engineer and can target more genomic locations than can HEs (Poster panel 5). From 2002 onwards, ZFNs became more widely used than HEs, especially as a research tool in various organisms, including flies, fish and plants (Urnov et al., 2010; Carroll, 2011). The first ZFN-modified mutant mouse models were described in 2010 by Carbery and co-workers via the direct injection of ZFNs that target and inactivate *Mdr1a*, *Jag1* and *Notch3* (Carbery et al., 2010). Nevertheless, the technical complexity of building ZFNs, and intellectual property restrictions, limited their widespread adaptability. TALENs, the next set of programmable nucleases, were developed in 2010 and overcame many of the limitations of HEs and ZFNs. TALENs were simpler, easier to build and could be used to target a greater number of genomic sites than could HEs or ZFNs, and thus were immediately adopted by hundreds of labs as research tools. The first mutant mouse models using TALENs were developed by Sung and co-workers in 2013 via the direct injection of TALENs that targeted *Pibf1* and *Sepw1* to inactivate them (Sung et al., 2013).

At the time when ZFNs and TALENs were being developed, each platform proved to be quite versatile and superior to the previously available genetic engineering tools. Then came the development of the CRISPR/Cas9 genome editing tool in late 2012 and early 2013 (Jinek et al., 2012; Cong et al., 2013; Mali et al., 2013) (Poster panel 5). A series of papers from multiple groups, published within a few months of each other, demonstrated that dsDNA breaks at specific sites in the genome could be generated with very high efficiency in mammalian cells by using guide RNAs complementary to the target site and the Cas9 nuclease (Jinek et al., 2012, 2013; Mali et al., 2013; Cong et al., 2013; Cho et al., 2013). Within just a few months, some groups demonstrated that the RNA-guided Cas9 nuclease system could be used to rapidly generate mutant mouse models (Shen et al., 2013; Wang et al., 2013). Since then, the RNA-guided Cas9 nuclease system has almost completely superseded all other technologies for genome editing. A direct comparison of the RNA-guided Cas9 nuclease system with the previous nuclease-based platforms (HEs, ZFNs and TALENs) clearly shows that it has several advantages (Sander and Joung, 2014; Porteus, 2015; Woolf et al., 2017). These include its simplicity of use, lower cost and higher efficiency. The RNA-guided Cas9 nuclease system is constantly being improved to make it increasingly efficient and versatile, including optimizing and improving the efficiency of existing Cas nucleases (Kleinstiver et al., 2016; Slaymaker et al., 2016), and the development of novel Cas nucleases (Shmakov et al., 2015; Zetsche et al., 2015). The RNA-guided Cas9 nuclease system is considered a ‘disruptive’ technology because it is quickly making previously well-established and fully developed technologies outdated. In recent years, researchers have come to prefer this approach over ES-cell-based gene-targeting methods (Burgio, 2018; Skarnes, 2015) because RNA-guided Cas9 nuclease approaches are relatively quicker, less expensive and less cumbersome.

The versatility of the RNA-guided Cas9 nuclease system allows researchers to engineer and edit the genome in ways that were previously not possible using non-nuclease-based approaches (Poster panel 5). This includes the ease and speed with which researchers can induce a footprint-free point mutation (Box 1) (Inui et al., 2014; Gurumurthy et al., 2016a). Many human disease conditions are caused by subtle genetic changes, such as point mutations, or by the addition or deletion of a few nucleotides (Gonzaga-Jauregui et al., 2012). Developing animal models of such subtle genetic changes, by using ES-cell-based targeting approaches, inevitably requires the addition of other genetic elements near the vicinity of the genetic change [such as a drug selection marker (neomycin or puromycin) and recombinase elements (such as *loxP* or *FRT* sites)]. By contrast, the RNA-guided Cas9 nuclease system can generate animal models with subtle genetic changes with high precision, rapidly, efficiently and without leaving any residual genetic alterations. Compared to previous methods, this capability represents a significant advance in murine genome editing for human disease modeling. The RNA-guided Cas9 nuclease tool has also facilitated the generation of multiple mutant mouse models in a single experiment by inducing dsDNA breaks at multiple target sites, resulting in several different gene disruption models (Wang et al., 2013). The RNA-guided Cas9 nuclease system also enables the generation of mutant mouse models on genetic backgrounds that were not amenable to being genetically manipulated with earlier approaches, such as the immunodeficient NOD/Scid-ILgamma (NSG) strain (Li et al., 2014). The RNA-guided Cas9 nuclease system has also become a powerful tool for both forward and reverse genetics (Gurumurthy et al., 2016c), generating models that are relevant for many diseases, including cancer (Platt et al., 2014). Several recent review articles discuss the Cas9-nuclease-generated mouse models for different disease types, including for cancer (Mou et al., 2015; Roper et al., 2017), cardiovascular diseases (Miano et al., 2016), neurodegenerative diseases (Yang et al., 2016) and kidney diseases (Higashijima et al., 2017). In addition, several reviews on Cas9-nuclease-generated models have been recently published that discuss their human disease relevance (Dow, 2015; Tschaharganeh et al., 2016; Cai et al., 2016; Yang et al., 2016; Birling et al., 2017).

Despite its advantages, the RNA-guided Cas9 nuclease system poses challenges, such as mosaicism (Yen et al., 2014) and off-target effects. If one of the two haploid genomes in the one-cell-stage zygote is not cleaved before the zygote divides, or if Cas9 activity persists at the two-cell or later stages, additional mutant alleles can be generated, resulting in more than three mutant alleles in the developing offspring. Consequently, as many as six or more types of alleles were detected in one founder (G0) mouse (Li et al., 2013). It is therefore essential to genotype F1 offspring to identify a desired mutant allele. This mosaicism can also be considered an advantage because multiple different alleles can be segregated and used as separate mutant models. For example, the same founder mouse could contain a complete insertion deletion (indel) allele and the foreign cassette knock-in allele; each can be used for different research applications. Because the Cas9 target sequence is only 23 nucleotides long, including the protospacer adjacent motif, it is likely that imperfect target-matching sequences are present elsewhere in the genome that contain one or a few mismatches. Cas9 can potentially bind to such imperfect target sites and thus generate dsDNA breaks and indels at those sites. Indel mutations in off-target sites can have confounding effects in mouse phenotyping experiments. However, off-target effects are not considered a major concern because they: (i) are generally negligible in mice (Iyer et al., 2015); and (ii) can be segregated during mouse breeding. Another recent study, now retracted, reported the presence of high rates of off-target effects in Cas9 engineered mice (Schaefer et al., 2017); however, this report's experimental design and interpretations have been questioned by the scientific community (Kim et al., 2018; Lescarbeau et al., 2018; Nutter et al., 2018; Wilson et al., 2018).

A current challenge to the broader use of RNA-guided Cas9 nuclease is the inability to use it to insert large fragments of DNA reliably and efficiently. Because most genetic-engineering approaches in mice involve the insertion of engineered DNA cassettes, efforts are underway to improve the ‘knock-in’ capabilities of this system. While a few RNA-guided Cas9 nuclease strategies have been modified to support the insertion of new cassettes (Aida et al., 2015; Maruyama et al., 2015; Sakuma et al., 2016), including a strategy that combines PITT and RNA-guided Cas9 nuclease approaches (Quadros et al., 2015), none has yet been successfully adapted for the routine engineering of the mouse genome. A report from Ohtsuka's group, which used long single-stranded DNA (lssDNA) donors (generated via *in vitro* transcription and reverse transcription), demonstrated that lssDNAs could serve as efficient donors for insertion at the Cas9 cleavage sites (Miura et al., 2015). Another report, which used lssDNAs purified from nicked plasmids to create rat knock-in models, also demonstrated that the lssDNA donor strategy could be a reliable approach for creating insertion alleles (Yoshimi et al., 2016). More recent reports show that co-injecting lssDNA donors with commercially available CRISPR ribonucleoprotein complexes (instead of the previous formats of *Cas9* mRNA and sgRNAs), offers a highly robust and efficient strategy for insertion alleles in a method termed *Easi-*CRISPR (efficient additions with ssDNA inserts-CRISPR) (Quadros et al., 2017; Miura et al., 2017).

RNA-guided Cas9 nuclease reagents have also been delivered into zygotes via electroporation of RNA and/or of ribonucleoproteins (Chen et al., 2016; Hashimoto and Takemoto, 2015; Qin et al., 2015). The ability to deliver RNA-guided Cas9-nuclease gene-editing reagents into several zygotes at once overcomes the need to inject each individual zygote, one at a time, and greatly simplifies the process of generating mouse models. Furthermore, electroporation is less damaging to embryos than microinjection (Chen et al., 2016; Hashimoto and Takemoto, 2015; Qin et al., 2015). Another advance in delivering the RNA-guided Cas9 nuclease system is a method called GONAD (genome editing via oviductal nucleic acids delivery). This procedure delivers Cas9 reagents to embryos in the oviduct using electroporation (Takahashi et al., 2015; Gurumurthy et al., 2016b; Sato et al., 2016; Ohtsuka et al., 2018). Unlike standard approaches, this method does not require any of the three major steps of animal transgenesis: zygote isolation from a female donor; *ex vivo* handling of zygotes (involving either microinjection or electroporation); and the transfer of zygotes to a pseudopregnant female mouse. This approach requires surgical skills that are equivalent to performing the oviductal transfer of embryos. The GONAD method can be used to generate knockout mice (Takahashi et al., 2015), and, by using the so-called improved-GONAD (*i-*GONAD), more complex animal models, such as knock-ins and large-deletion models, can be generated at an efficiency similar to the microinjection-based methods (Ohtsuka et al., 2018). The *i-*GONAD method also uses only a third of the mice used in standard microinjection or in *ex vivo* zygote electroporation methods (Ohtsuka et al., 2018). These methods need not be limited to centralized facilities, sophisticated equipment or highly skilled technical personnel. It is thought that the technical advances such as *Easi-*CRISPR and *i-*GONAD have the potential to entirely reshape the traditional route of generating modified alleles in mice if the techniques are widely adopted by many research groups and by transgenic core facilities (Burgio, 2018).

## Concluding remarks and future perspectives

Recent technological breakthroughs have enabled very rapid changes in the way we generate genetically altered mouse models. Most notably, the RNA-guided Cas9 nuclease system is assuming a key role in shaping this new technological landscape. While the use of the RNA-guided Cas9 nuclease system has transformed and eclipsed traditional transgenic technologies in many ways, challenges remain, including the inability to insert large DNA constructs to generate a knock-in mouse (Box 1) with reporter, conditional or humanized alleles, or to engineer chromosomal rearrangements and other complex alleles easily, routinely and efficiently.

Genetic manipulation also underpins the ongoing efforts to elucidate the functional roles of every gene in the mouse genome, as a first step to understanding the role of ‘disease alleles’ identified by the exome and genome sequencing of human patients. Genomic and precision medicine depends on our ability to differentiate benign from pathogenic variant alleles, and disease-causing alleles from the longer list of disease-associated ones. Genetic manipulation of the mouse genome is thus essential for understanding gene function and for uncovering the genetic and molecular basis of human disease, leading to improved diagnostic accuracy, development of targeted therapeutics and the implementation of effective prevention strategies.
